# Disentangling longitudinal relations between physical activity, work-related fatigue, and task demands

**DOI:** 10.1007/s00420-015-1054-x

**Published:** 2015-05-08

**Authors:** Juriena D. de Vries, Brigitte J. C. Claessens, Madelon L. M. van Hooff, Sabine A. E. Geurts, Seth N. J. van den Bossche, Michiel A. J. Kompier

**Affiliations:** Behavioural Science Institute, Radboud University, P.O. Box 9104, 6500 HE Nijmegen, The Netherlands; Department of Work, Health & Care, TNO, Leiden, The Netherlands

**Keywords:** Work-related fatigue, Physical activity, Task demands, Longitudinal research, Physical activity norm

## Abstract

**Purpose:**

This longitudinal study examined ‘normal’, ‘reversed’, and ‘reciprocal’ relationships between (1) physical activity and work-related fatigue; and (2) physical activity and task demands. Furthermore, the effects of across-time change in meaningful physical activity groups on levels of employees’ work-related fatigue and task demands were studied. These groups were based on employees’ compliance with the international physical activity norm.

**Methods:**

Two waves with a one-year time lag of a national representative survey on the quality of work, health, and well-being among Dutch employees were used (*N* = 2275). Longitudinal effects were tested using Structural Equation Modelling. Meaningful physical activity groups were compared using group-by-time analysis of covariance.

**Results:**

Support was found for reciprocal relations between physical activity and work-related fatigue. It was found that an increase in physical activity is associated with a decrease in work-related fatigue over time and that an increase in work-related fatigue is associated with a decrease in physical activity over time. No significant longitudinal relations were found between physical activity and task demands. Employees whose compliance with the physical activity norm changed over time showed fairly stable levels of work-related fatigue and task demands.

**Conclusions:**

The current findings provide evidence for the potential role of physical activity in the prevention and reduction in work-related fatigue. However, results also indicate that fatigued workers, who would benefit most from physical activity, are less physically active. Our results further indicate that relying on changes in compliance to the physical activity norm may not be the most suitable way to examine changes in work-related fatigue.

## Introduction

There is a growing body of evidence that physical activity is an effective remedy against mental health problems (Conn 2010; Cooney et al. 2013). As a substantial proportion of mental health problems is work related (estimated at 22 %; Eurofound 2012; Niedhammer et al. 2014), it is valuable to examine the potential of physical activity to reduce such problems. Although previous studies do point to negative associations between physical activity and, for example, work-related fatigue (e.g. Bernaards et al. 2006; Carson et al. 2010), job stress (Van Rhenen et al. 2005), and burnout (Gerber et al. 2013; Jonsdottir et al. 2010), insight into the role of physical activity in the prevention and reduction in these types of problems can be advanced in at least three ways.

First, the ‘bi-directional’ relationships between physical activity and work-related mental health need to be addressed. Existing studies almost exclusively focused on the question how physical activity affects work-related mental health (e.g. Bernaards et al. 2006; Carson et al. 2010; Jonsdottir et al. 2010), and ignored the possibility that employees’ work-related mental health status may also influence the amount of physical activity they engage in. This is unfortunate, as it may be expected that employees who report high levels of work-related fatigue lack the resources to engage in regular physical activity. In other words, it is likely that work-related mental health and physical activity mutually affect each other.

Secondly, it has been widely established that adverse work characteristics play a key role in the aetiology of work-related mental health problems (e.g. Kompier 2003; De Lange et al. 2003). Therefore, to obtain a complete picture of the potentially beneficial role of physical activity for employee health and well-being, paying attention to their work environment is of vital importance. Although empirical evidence shows that (certain combinations of) work characteristics are related to employees’ physical activity level (Fransson et al. 2012; Kouvonen et al. 2013), the directionality of these associations is still not well understood.

Third, previous research on the associations between physical activity, work, and mental health was mainly cross sectional in nature, and the few longitudinal studies that exist did not take into account that for some employees a change in their level of physical activity may have been ‘meaningful’ (e.g. from considerably high levels to considerably low levels), whereas others report a stable (high or low) level of activity over time. It is therefore valuable to closely examine meaningful subgroups that differ in their initial levels and course of physical activity over time (i.e. ‘stability and change paradigm’; De Lange et al. 2002; Van Hooff et al. 2005). This paradigm can provide more insight in what changed levels of physical activity mean for levels of work-related mental health and work characteristics.

In this study, we aim to enhance insight into the association between physical activity and work-related mental health by addressing these three issues. To these purposes, we used a two-wave longitudinal full panel design with a one-year time interval of a survey on the quality of work, health, and well-being among Dutch employees. We focused on work-related fatigue as an indicator of work-related mental health problems, as work-related fatigue is the most commonly reported element of burnout (Maslach et al. 2001) and prevalent among the working population (i.e. estimated at 18 % in Europe; Milczarek et al. 2009). In addition, based on Karasek’s (1979) Job-Demands-Control Model, we focused on ‘quantitative task demands’ as an indicator of work characteristics. Task demands refer to the degree to which work requires employees’ effort (Hockey 2013), for instance, working fast and performing a lot of work. Finally, we defined physical activity as an activity that requires (at least moderate intensity) physical effort (WHO 2010).

### Physical activity and work-related fatigue

Although physical activity requires physical energy and physical recovery (Ament and Verkerke 2009), it can also deliver ‘mental’ energy and reduce feelings of (work-related) fatigue (e.g. Bültmann et al. 2002; Lindwall et al. 2013). The exact working mechanisms underlying these observed associations are still unclear (Puetz and Herring 2013). Both biological and psychological hypotheses have been proposed. Concerning the former, endorphin or monoamine hypotheses state that physical activity results in changes in certain neurotransmitters (e.g. endorphin) that are associated with feelings of energy, but the evidence is still weak (Dishman and O’Connor 2009). Further, by means of regular physical activity, the body is ‘toughened up’ and is better able to handle (psychological) stress (Sothman et al. 1996). This results in lower bodily reactions due to (work) stress (i.e. lower stress reactivity; Wipfli and Ramirez 2013) and faster bodily recovery after being exposed to (work) stress (i.e. faster stress recovery; Spalding et al. 2004). Several other biological processes have been proposed as well, see for an overview Dishman et al. (2006).

With regard to psychological hypotheses, it has been proposed that physical activity increases people’s self-efficacy (Craft 2005), generates positive feelings about the self (Feuerhahn et al. 2014), and creates a more positive body image (Campbell and Hausenblas 2009). Physical activity may also generate energy by providing people the opportunity to distract themselves from negative stimuli, such as rumination about work (i.e. ‘psychological detachment’, Sonnentag 2012; distraction hypothesis, Leith 1994), and instead, shift towards more pleasant stimuli (Tian and Smith 2011).

In line with these proposed beneficial effects, cross-sectional studies indeed show negative associations between physical activity and work-related fatigue (Carson et al. 2010; Mollart et al. 2013). The few available longitudinal (Bernaards et al. 2006; Lindwall et al. 2013) and intervention studies (e.g. Gerber et al. 2013; Proper et al. 2003; Tsai et al. 2013; Van Rhenen et al. 2005) show comparable relationships. Also, diary studies indicate that physical activity can decrease work-related fatigue on a daily level (Nägel and Sonnentag 2013; Rook and Zijlstra 2006). Thus, based on previous theory and empirical findings, we hypothesize:

#### **Hypothesis1a**

Higher physical activity levels are associated with lower levels of work-related fatigue one year later.

The opposite relationship between physical activity and work-related fatigue may exist as well: employees who experience high levels of work-related fatigue may be less physically active. Generally, fatigue is seen as an adaptive phenomenon: it is a signal to stop a certain task (before damage occurs) and is therefore associated with people having a lower tendency to start or complete tasks, in particular when a task requires a high level of effort (Hockey 2013). As physical activity is effortful, it can be assumed that work-related fatigue will negatively affect the extent to which employees engage in this type of activity. In support of this assumption, scarce available empirical evidence shows that employees experiencing higher levels of work-related fatigue reported lower levels of physical activity (Ahola et al. 2012; Gorter et al. 2000). Based on the rationale that fatigue is associated with a tendency to avoid physical activity due to the effort this requires, we propose:

#### **Hypothesis1b**

Higher levels of work-related fatigue are associated with lower levels of physical activity one year later.

### Physical activity and task demands

Employees’ level of task demands at work may negatively affect the extent to which they engage in physical activity. First, high task demands may deplete personal (e.g. self-regulatory, Nägel and Sonnentag 2013; Sonnentag and Jelden 2009) and other resources (e.g. time, due to commuting or long working hours) that are needed to engage in physical activity. Furthermore, based on the perseverative cognition hypothesis (Brosschot et al. 2006), it can be expected that employees with higher task demands at work stay cognitively preoccupied with work during off-job time, which prolongs their physiological activation after work. It has been found that this inability to cognitively ‘switch off’ from work is associated with less personal control over leisure time (Cropley and Purvis 2003), making it more difficult to engage in physical activity. Indeed, the scarce available research shows a negative association between task demands and levels of physical activity (Fransson et al. 2012; Kouvonen et al. 2013; Payne et al. 2002). Based on these theoretical notions and empirical findings, we expect:

#### **Hypothesis2a**

Higher levels of task demands are associated with lower levels of physical activity one year later.

One could also argue that employees’ physical activity level influences perceived task demands at work. An explanation for this association is that physical activity enhances individuals’ (physiological, psychological, and cognitive) health, and hence increases employees’ ability to handle demands during the workday, as they require less effort (cardiovascular fitness hypothesis, e.g. Colcombe and Kramer 2003). In other words, physical activity may lead to increased (physiological, psychological, and cognitive) capacity to cope with the demands at work. Indeed, research indicates that physical activity can contribute to employees’ capacity to perform their assigned tasks (Arvidson et al. 2013). Research has also shown that regular physical activity is associated with mastery experiences and increases in self-efficacy (Craft 2005). Increased self-efficacy may be transferred to the work domain, resulting in employees feeling more competent to meet the task demands at work (Feuerhahn et al. 2014; Rook and Zijlstra 2006). Consequently, they may experience their tasks as less demanding. Thus, based on the idea that physical activity may increase employees’ capacity to cope with work demands and therefore causes a shift towards a more positive evaluation of these demands, we hypothesize:

#### **Hypothesis2b**

Higher levels of physical activity are associated with lower levels of task demands one year later.

### Meaningful subgroups based on physical activity norm

To get further insight into the role of physical activity in relation to work-related mental health and work characteristics, it is worthy to examine ‘meaningful’ subgroups that differ in their initial levels and course of physical activity over time (cf. De Lange et al. 2002; Van Hooff et al. 2005). Therefore, in the present study, we examine two groups of employees who differ in their starting points and courses over time regarding their engagement in physical activity. To create these meaningful subgroups, we rely on the international norm for physical activity developed by the World Health Organization (World Health Organization [WHO] 2010), which states that people of 18 years or older should engage in at least 30 min of moderate-intensity physical activity on at least 5 days a week (in bouts of minimally 10 min a time) to stay healthy (Hildebrandt et al. 2010). The two groups comprise the following: (1) those employees who do not comply with the physical activity norm at the first time, but do so at the second time (i.e. ‘upward’ indicating a beneficial change); and (2) those employees who comply with the exercise norm at the first time, but do not at the second time (i.e. ‘downward’ indicating an unfavourable change). We expected—in accordance with previous hypotheses—that compliance with the physical activity norm is related to lower levels of work-related fatigue and task demands:

#### **Hypothesis3a**

Employees in the upward physical activity group (i.e. ‘low–high’) report a decrease in work-related fatigue and task demands 1 year later.

#### **Hypothesis3b**

Employees in the downward physical activity group (i.e. ‘high–low’) report an increase in work-related fatigue and task demands 1 year later.

## Methods

### Sample

This study was based on a two-wave full panel design with a one-year time lag. The participants were part of the TNO-Netherlands Working Conditions Cohort Study in 2008 and 2009 (NWCCS; Koppes et al. 2010), a survey focused on quality of work, health, and well-being of Dutch employees (self-employed were excluded from the sampling framework). A total of 7909 employees (76.10 % of the initial approached employees in 2008) filled out the questionnaire in both 2008 and 2009. We selected employees who worked fulltime (≥36 h a week), to ensure a sufficient exposure to task demands at work. This restriction reduced our sample size to 3583 employees. Furthermore, we excluded employees who worked in physically demanding jobs, because these jobs generally require ‘unhealthy physical activity’, such as lifting and pushing, which has already been found to be related to unfavourable health outcomes (e.g. Trinkoff et al. 2001). Hence, we only included employees who answered ‘no’ to the question ‘Do you perform work in which you have to put strength, such as pushing, lifting, pulling, and hauling, or do you use tools and equipment in which you have to put strength?’ (1 = yes, regularly, 2 = yes, sometimes, 3 = no). This exclusion criterion further reduced our sample size to 2275 employees. Of this final sample (*N* = 2275, 28.8 % of the original sample), 75.3 % were male (*M*_age_ 45.8, SD = 10.0) and 24.7 % female (*M*_age_ = 39.9, SD = 11.4). This distribution differed from the original sample in which 48.3 % were male (*M*_age_ 46.3, SD = 10.9) and 51.7 % were female (*M*_age_ 42.9, SD = 11.2). Mean working hours of the final sample were 38.4 (SD = 3.1) and mean working days were 4.9 (SD = 0.5). The employees of the final sample were mainly well educated (60.3 % higher professional education), and this differed from the original sample (42.8 % higher professional education). Selected employees primarily worked in the area of business services (19.3 %), public administration (17.5 %), industry (14.6 %), and education (9.9 %). These figures were comparable with those in the original sample, except that much more employees in the original sample worked in a healthcare setting (23.0 %) compared to the final sample (8.2 %).

### Materials

Task demands were measured with a four-item scale (e.g. ‘Do you have to work fast?’; 1 = never, 2 = sometimes, 3 = often, 4 = always) that was derived from a Dutch version of the Job Content Questionnaire (JCQ; Houtman 1995; Karasek et al. 1998). The reliability of the scale was high for both waves (Cronbach’s *α* = 0.86 in 2008 and 0.85 in 2009, respectively).

Physical activity was assessed with the following question: ‘On how many days a week are you normally physically active during at least 30 min a day (only count physical activity that is equally demanding as brisk walking or biking. Activities shorter than 10 min do not count)*—*during your work and free time together?’ Participants indicated how many days they complied with a minimum of 30 min of physical activity (0–7 days). This item was based on international standards for physical activity (World Health Organization [WHO] 2010), which state that people ≥18 years of age should engage in at least 30 min of moderate-intensity physical activity minimally 5 days a week (in bouts of minimally 10 min a time) to stay healthy.

Work-related fatigue was measured with the five-item ‘exhaustion’-subscale of the Dutch version of the Maslach Burnout Inventory (Utrechtse Burnout Scale [UBOS] Schaufeli and Van Dierendonck 2000). A typical item is: ‘I feel burned out from my work’ (0 = never, 6 = every day). The reliability of the scale was high for both waves (Cronbach’s *α* = 0.87 in 2008 and 0.88 in 2009, respectively).

Control variables, age, gender, education, working overtime and working irregular hours, measured at T1, were included as control variables. Gender was coded as 1 = male and 2 = female. Education was coded as 1 = low; 2 = intermediate; 3 = high professional education. Working overtime was assessed as overtime hours, using the following question: ‘On average, how many hours a week do you work overtime?’ (paid and unpaid work; include work you execute at home; don’t include your commuting time). For working irregular hours, a variable was computed, in which employees who had no irregular work were classified as ‘1’, and employees who worked at night, in the evening, in the weekend, or had shift work were classified as ‘2’.

### Statistical approach

Descriptive statistics (means, standard deviations, percentages, and correlations) were calculated in order to study the prevalence of task demands, physical activity, and work-related fatigue for 2008 (T1) and 2009 (T2). Additionally, it was observed whether compliance with the physical activity norm is related to (high levels of) work-related fatigue and task demands. Next, two steps were taken to test our hypotheses.

#### Across-time relationships

To test Hypotheses 1a and 1b, Structural Equation Modelling (SEM) was performed using LISREL version 9.1 (Jöreskog and Sörbom 1993). SEM was used because this technique allowed us to test reciprocal relationships between constructs. To investigate the associations between physical activity and work-related fatigue (Hypotheses 1a and 1b), four models were compared to each other. The first model (M1; no causation) included lagged effects from physical activity at T1 to physical activity at T2, and from work-related fatigue at T1 to work-related fatigue at T2. Age, gender, education, working overtime, and working irregular hours were added as covariates to this model and were modelled to be related to physical activity and work-related fatigue at T1. The second model (M2; normal causation) resembled M1, but also included a path from physical activity T1 to work-related fatigue T2. The third model (M3; reversed causation) resembled M1, but now included a ‘reversed’ path from work-related fatigue T1 to physical activity T2. The fourth model (M4; reciprocal causation) resembled M1 and additionally included the paths of M2 and M3 so that reciprocal relationships between physical activity and work-related fatigue were investigated. The fit of the four models was compared using Chi-square difference tests, the comparative fit index (CFI), the non-normed fit index (NNFI), the adjusted goodness-of-fit index (AGFI), and the root mean square error of approximation (RMSEA) (Bentler and Bonnett 1980). Model fit was considered acceptable if the NNFI, CFI, and the AGFI were ≥0.90 and RMSEA was ≤0.08 (Marsh et al. 2004).

To test Hypotheses 2a and 2b (associations between task demands and physical activity), similar analytical steps were used, meaning that again four models were compared with each other. As we entered variables measured on T1 as a predictor into the analyses, we controlled for T1—T2 stability effects. As a result, the results of SEM reflect changes between T1 and T2 of, respectively, physical activity, task demands, and work-related fatigue.

#### Subgroup analyses

We investigated whether changed levels of compliance with international standards of physical activity were related to accompanying changes in levels of task demands and work-related fatigue (**Hypothesis3a** and **3b**). Therefore, for T1 and T2, two groups were created based on employees’ physical activity level. If employees were physically active for at least 30 min a day on <5 days a week, they were classified as ‘low’ (i.e. not meeting the physical activity norm). If employees were physically active at least 30 min on 5 days or more, they were classified as ‘high’ (i.e. meeting the physical activity norm).

To test the hypotheses, employees were incorporated in a ‘low–high’ (non-compliance with the norm at T1 and compliance with the norm at T2) or ‘high–low’ (compliance with the norm at T1 and non-compliance with the norm at T2) group. After that, we conducted a 2 (group: ‘low–high’ vs. ‘high–low’) × 2 (time: T1 vs. T2) ANCOVA with repeated measures on time (RM–ANCOVA) for continuous measures of work-related fatigue and task demands, respectively, and focused on ‘group × time’, ‘time’, and ‘group’ effects. We controlled for age, gender, education, working overtime, and working irregular hours.

## Results

### Descriptive statistics

The means and standard deviations of study variables at both time points are presented in Table 1. On average, participants were physically active for at least 30 min on a moderate intensity on 4 days a week (T1: *M* = 3.98; T2: *M* = 4.09). Further inspection of our data revealed that 43.9 % complied with the physical activity norm at T1 and 45.9 % at T2. This is lower than a representative sample in the Netherlands, in which 58 % of the population complies with the physical activity norm (Hildebrandt et al. 2013). Most participants reported low levels of work-related fatigue [at T1, 12.4 % reported high (i.e. higher than the cut-off score of 2.2, see Schaufeli and Van Dierendonck 2000) levels of work-related fatigue, and at T2 this was 12.2 %]. Furthermore, participants displayed relatively high levels of task demands with a mean of 2.44 for T1 and a mean of 2.42 for T2, implying that most employees experienced task demands more frequently than ‘sometimes’.

**Table 1:** Means, standard deviations (SD), and correlations among study variables (*N* = 2275)

Variables	*M*/ Frequency	SD	Theoretical range	1	2	3	4	5	6	7	8	9	10
1. Gender^a^	1) 75.3 %		1 = male										
	2) 25.7 %		2 = female										
2. Age^a^	44.34	10.66	19–65	−0.24**									
3. Education^a^	1) 8.9 %		1 = low	0.05*	0.16**								
	2) 30.8 %		2 = intermediate										
	3) 60.3 %		3 = high										
4. Hours working overtime^a^	4.01	4.44	0–38	−0.08*	0.03	0.14**							
5. Irregular working hours^a^	1) 80.8 %		1 = no irregular hours	0.05*	−0.01	0.07**	0.24**						
	2) 19.2 %		2 = irregular hours										
6. Task demands T1	2.44	0.60	1–4	−0.01	0.00	0.20**	0.35**	0.08**					
7. Task demands T2	2.42	0.60	1–4	0.08	−0.03	0.21**	0.31**	0.06**	0.68**				
8. Work-related fatigue T1	0.95	1.01	0–6	0.05*	−0.03	0.08**	0.04	0.01	0.28**	0.22**			
9. Work-related fatigue T2	0.97	1.03	0–6	0.02	−0.04	0.07**	0.04	−0.01	0.24**	0.28**	0.67**		
10. Physical activity T1	3.98	2.14	0–7	0.03	0.09**	−0.02	−0.12**	−0.04	−0.06**	−0.07**	−0.08**	−0.08**	
11. Physical activity T2	4.09	2.10	0–7	0.03	0.13**	−0.03	−0.10*	0.00	−0.07**	0.08**	−0.09**	−0.08**	0.65**

* *p* < 0.05; ** *p* < 0.01

^a^Score at T1

Table 1 shows that the core variables under study remain relatively stable between T1 and T2, as indicated by T1–T2 correlations of 0.65 (for physical activity), 0.67 (for work-related fatigue), and 0.68 (for task demands). The pattern of correlations was in the expected direction of our hypotheses, both cross sectional and across-time. A closer examination of compliance with the physical activity norm in relation to high levels of work-related fatigue reveals that at T1, 13.8 % of employees who complied reported high work-related fatigue (≥2.2), compared to 10.8 % who did not comply. For T2, these figures were 13.0 and 11.4 %, respectively. Furthermore, at T1, 50.8 % of employees who complied with the physical activity norm reported high levels of task demands (i.e. a mean score of ≥2.5 was considered as ‘high’, indicating that demands were more frequently experienced than ‘sometimes’), compared to 44.7 % who did not comply. At T2, these figures were 50.3 and 41.9 %, respectively.

### Across-time relationships

**Fig. 1:**
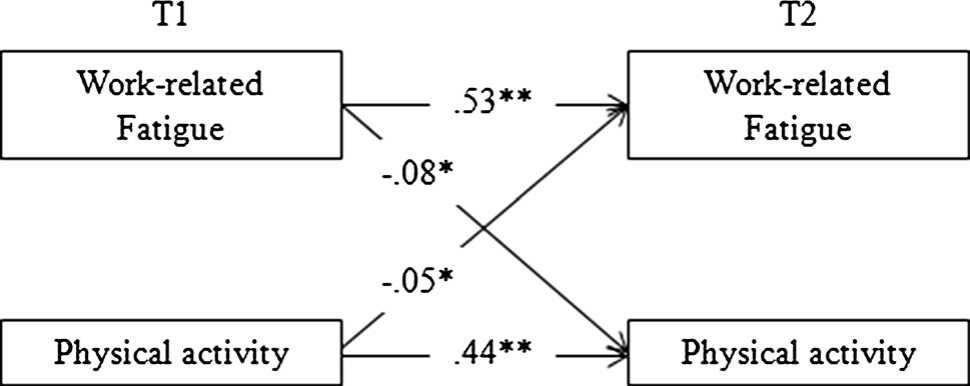
Reciprocal model (model 4) between work-related fatigue and physical activity (Hypothesis 1a and 1b), controlled for gender, age, education, working overtime, and irregular working hours. Standardized paths (*β*’s) are displayed. *p < 0.05; **p < 0.01

Fit indices of the four models that were compared to test Hypotheses 1a and 1b are presented in Table 2. Model 1 fitted the data well, with significant paths between work-related fatigue measured at T1 and at T2 (*β* = 0.53), and between physical activity measured at T1 and T2 (*β* = 0.44). Model 2 also fitted the data well and fitted significantly better than Model 1 (see Table 2 for model comparisons). This model reveals a significant negative association between physical activity T1 and work-related fatigue T2 (*β* = −0.05). Also Model 3 fitted the data well and fitted better than Model 1. This model shows a significant negative association between work-related fatigue T1 and physical activity T2 (*β* = −0.08). Table 2 shows that Model 4—including reciprocal associations between physical activity and work-related fatigue—has an acceptable fit as well and that this model fitted better than both Model 2 and Model 3. Consequently, we chose Model 4 as the best fitting model (see Fig. 1 for a graphical representation). This model shows that physical activity at T1 is associated with a decrease in work-related fatigue from T1 to T2, thus supporting **Hypothesis1a**. Further, the model shows that work-related fatigue is associated with a decrease in the level of physical activity from T1 to T2, thus supporting **Hypothesis1b**. Additionally, some covariates were significantly related to the constructs of interest at T1: working overtime (*β* = −0.09) and age (*β* = 0.05) were related to physical activity; and working overtime (*β* = 0.04) was related to work-related fatigue.

**Table 2:** Fit indices of structural equation models for the longitudinal associations between physical activity and work-related fatigue, and physical activity and task demands

Model	*χ* ^2^	*df*	NNFI	CFI	AGFI	RMSEA	Model comparison	Δ*df*	Δ*χ* ^2^
Physical activity and work-related fatigue (H1a and H1b)									
M1 (no causation)	57.48	12	0.92	0.97	0.98	0.04			
M2 (normal causation)	49.62	11	0.93	0.98	0.98	0.04	M1 versus M2	1	7.86**
M3 (reversed causation)	39.39	11	0.95	0.98	0.98	0.03	M1 versus M3	1	18.09**
**M4** (**reciprocal** **causation**)	**31.65**	**10**	**0.95**	**0.99**	**0.99**	**0.03**	M2 versus M4	1	17.97**
							M3 versus M4	1	7.74**
Physical activity and task demands (H2a and H2b)									
M1 (no causation)	58.05	12	0.93	0.98	0.98	0.04			
M2 (normal causation)	54.93	11	0.93	0.98	0.98	0.04	M1 versus M2	1	3.12
M3 (reversed causation)	57.12	11	0.93	0.98	0.98	0.04	M1 versus M3	1	0.93
M4 (reciprocal causation)	53.99	10	0.92	0.98	0.98	0.04	M2 versus M4	1	0.94
							M3 versus M4	1	3.13

The models are controlled for gender, age, education, working overtime, and working irregular hours

* *p* < 0.05; ** *p* < 0.01

Bold indicates best fitting model

To test Hypotheses 2a and 2b, again four models were compared. The fit indices and model comparisons of the four models are presented in Table 2. Model 1 fitted the data well, with significant paths from physical activity T1 to physical activity T2 (*β* = 0.44), and from task demands T1 to task demands T2 (*β* = 0.56). None of the extended models fitted better than Model 1, and therefore, **Hypothesis2a** (high levels of task demands are associated with lower levels of physical activity 1 year later) and **Hypothesis2b** (higher physical activity levels are associated with lower levels of task demands 1 year later) were not supported.

#### Subgroup analyses (Hypotheses 3a and 3b)

The estimated means and standard deviations of work-related fatigue and task demands for the different physical activity groups are presented in Table 3. The means and standard deviations of the stable physical activity groups (i.e. compliance at both times and non-compliance at both times) are also depicted in Table 3 to provide a complete picture of what stable and changed physical activity levels mean for work-related fatigue and task demands. As can be seen in Table 3, most participants displayed fairly stable physical activity levels, and relatively few participants changed with regard to compliance with the physical activity norm: 12.1 % of participants showed an upward change (*n* = 258) and 10.6 % showed an downward change (*n* = 225). The RM–ANCOVA revealed that there was no significant interaction between ‘group’ and ‘time’ for work-related fatigue [*F* (1,476) = 0.74, *p* = 0.391] and task demands [*F* (1,476) = 0.09, *p* = 0.662], meaning that changes in levels of work-related fatigue and task demands over time did not differ between the upward and downward physical activity group. The RM–ANCOVA also showed no effect of ‘time’ on work-related fatigue [*F* (1,476) = 0.02, *p* = 0.893] and task demands [*F* (1,476) = 0.07, *p* = 0.793], meaning that levels of work-related fatigue and task demands did not change over time. No effect of ‘group’ was found [*F* (1,476) = 0.04, *p* = 0.850], meaning that the groups did not differ on their mean levels of work-related fatigue and task demands. All in all, no support was found for **Hypotheses 3a** and **3b**.

**Table 3:** Means and standard deviations of work-related fatigue and task demands for the different meaningful physical activity groups, adjusted for age, gender, education, working overtime, and irregular working hours

Physical activity group	*n* ^a^	Work-related fatigue				*D* ^d^	Task demands				*D*
		T1		T2			T1		T2		
		*M*	SD	*M*	SD		*M*	SD	*M*	SD	
Low T1–low T2^b^ (stable low)	913	1.04	0.91	1.05	0.93	0.01	2.49	0.52	2.47	0.49	−0.04
High T1–high T2^c^ (stable high)	724	0.84	1.17	0.90	1.20	0.05	2.43	0.65	2.39	0.65	−0.06
Low T1–high T2 (upward change)	258	0.98	1.03	0.95	1.06	−0.03	2.38	0.56	2.39	0.56	0.02
High T1–low T2 (downward change)	225	0.90	1.02	0.88	1.05	−0.02	2.39	0.56	2.42	0.56	0.05

* *p* < 0.05; ** *p* < 0.01

^a^155 missing values; ^b^ ‘Low’ = not complying with the physical activity norm (i.e. <5 days a week 30 min of moderate-intensity physical activity); ^c ^‘High’ = complying with the physical activity norm (i.e. ≥5 days a week 30 min of moderate-intensity physical activity); ^d^ Cohen’s *D* effect size for the mean difference between T1 and T2

## Discussion

In this study, we examined longitudinal relationships between physical activity, work-related fatigue, and task demands. Our goal was threefold. First, we examined possible bi-directional relationships between physical activity and work-related fatigue (Hypotheses 1a and 1b). Secondly, we investigated whether employees’ task demands and physical activity level mutually influence each other (**Hypotheses 2a** and **2b**). Finally, we addressed the effects of change in employees’ adherence with the international physical activity norm in relation to work-related fatigue and task demands (**Hypotheses 3a** and **3b**). Table 4 presents an overview of support levels for this study’s hypotheses.

**Table 4:** Synthesis of evidence

Hypotheses		Longitudinal support
H1a	Higher levels of physical activity → lower levels of work-related fatigue	+
H1b	Higher levels of work-related fatigue → lower levels of physical activity	+
H2a	Higher levels of task demands → lower levels of physical activity	–
H2b	Higher levels of physical activity → lower levels of task demands	–
H3a	Upward physical activity group → decrease in work-related fatigue and task demands	–
H3b	Downward physical activity group → increase in work-related fatigue and task demands	–

In accordance with previous studies (e.g. Bültmann et al. 2002; Lindwall et al. 2013), we found that higher levels of physical activity were related to lower levels of work-related fatigue one year later (**Hypothesis1a**), although the size of this effect was relatively small. It should be noted, though, that longitudinal effects are always smaller and more difficult to detect than cross-sectional ones (Ford et al. 2014). Also, in this study, we controlled for the level of physical activity and work-related fatigue at T1 in our SEM. As these constructs were rather stable over time (across-time correlations *r* = 0.65 for physical activity and *r* = 0.67 for work-related fatigue), a large proportion of the variance in physical activity and work-related fatigue was already accounted for by the same indicator measured 1 year earlier. This means that the proportion of variance left to be explained was rather small. The association between physical activity and work-related fatigue was not supported by our subgroup analyses, which showed that a change in compliance with the physical activity norm (**Hypothesis3a** and **3b**) was not related to accompanying changes in work-related fatigue. This discrepancy in findings may be attributed to insufficient contrast between the two physical activity change groups. A closer examination of the data revealed that, in both groups, a notable proportion of participants reported just one or two days change in physical activity (i.e. 42.7 % in the ‘upward’ change group, and 47.6 % in the ‘downward’ change group changed one or two days). As a result, it may be difficult to detect intergroup differences in the development of work-related fatigue over time. Thus, within our relatively stable sample, distinguishing between subgroups based on changes in the physical activity norm may not be a sensitive enough method to capture differences in patterns of work-related fatigue over time.

Our results also support the hypothesis that employees’ level of work-related fatigue was negatively related to engaging in physical activity (**Hypothesis1b**). This could imply that being tired from work is a decisive factor for employees in whether or not to engage in physical activity. This result also implies that even the relatively low levels of work-related fatigue that were experienced by the employees in this study may already interfere with their physical activity levels. Finding reciprocal relations between physical activity and work-related fatigue may point to a downward spiral, in which more work-related fatigue is related to lower physical activity, which in turn relates to even higher levels of work-related fatigue.

Contrary to the few previous studies that addressed this association (Feuerhahn et al. 2014; Fransson et al. 2012; Kouvonen et al. 2013; Payne et al. 2002; Sonnentag and Jelden 2009), we did not find a longitudinal negative association between task demands and physical activity (**Hypothesis2a**). Our result may be explained by the fact that task demands especially affect physical activity during leisure time and not during work time (Fransson et al. 2012; Kouvonen et al. 2013), and affect rather activities that are voluntary than compulsory. Unfortunately, due to the measurement of physical activity in this current study, it was unknown whether employees’ physical activity entailed (voluntary) sport activities during leisure time, or that it was part of compulsory activities during daily life (e.g. at work or during household chores). The latter types of activities are often obligatory and part of daily routines and will thus not be easily skipped, while (voluntary) sports activities during leisure time may be more easily omitted if one’s resources are depleted due to high task demands.

Also, no support was found for the idea that higher levels of physical activity are related to lower levels of task demands over time (**Hypothesis2b**). Similarly, the upward and downward physical activity groups did not show changes in task demands over time (Hypotheses 3a and 3b). An explanation for these findings may be that task demands are partly ‘inherent’ to the job and thus cannot easily be changed. It may also be that changes in physiological, psychological, and cognitive health that can develop within a one-year time lag are too small to induce a response shift in the evaluation of task demands. Furthermore, it may be that not all types of physical activity impact task demands. For instance, one can imagine that sport activities result in mastery experiences (Craft 2005), which reduce perceived task demands, due to an increase in self-efficacy in the work domain. For other physical activities, such as physical household activities, this association may not be found, because these may not be associated with mastery experiences. Unfortunately, again referring to the measurement of physical activity in this current study, we cannot distinguish between these different types of physical activity.

## Limitations and suggestions for future research

There are seven issues concerning the current study that deserve attention. First, we exclusively relied on self-report measures in the present study. Some consider this to be a limitation, because it would result in an overestimation of the associations among variables due to common method variance. Based on his study of the potential problem of common method variance, Spector (2006) nonetheless concluded that ‘the popular position suggesting that common method variance automatically affects variables measured with the same method is a distortion and oversimplification of the true state of affairs’ (p. 221). Besides, internal states, such as work-related fatigue, can best be mapped by means of reports by those who are involved in these experiences. This notwithstanding, it would be valuable for future studies to combine self-report measures of physical activity with more objective methods, such as accelerometers or actigraphy (Prince et al. 2008).

Second—and related to the first issue—the self-report measurement of physical activity deserves attention. In general, people often over- or underestimate their true physical activity level, for instance due to recall bias or social desirability (Prince et al. 2008). Therefore, it is likely that self-report measures in this current study do not precisely reflect employees’ actual physical activity levels. In addition, participants in the current study were asked ‘on how many days a week they were physically active for at least 30 min at a moderate intensity’. As a result, the exact total duration and frequency of employees’ physical activity were unknown (e.g. when someone answers ‘3 days’, it could be exactly 90 min, but also more than this amount). Also, the exact intensity of the physical activity was not known. We only knew that the physical activity was at least of moderate intensity. Intensity is important, because it may affect work-related fatigue in different ways. For instance, it has been found that physical activity at a high intensity heightens someone’s fatigue and may even lead to exhaustion (Loy et al. 2013), whereas low (Puetz et al. 2008)-to-moderate intensity (Salmon 2001) physical activity levels are related to lower fatigue. Furthermore, the type of physical activity was unknown (e.g. non-aerobic training, physical activity as part of daily life, or as sport activity). Different types of physical activity could have distinct effects on work-related fatigue. There are reasons to believe that physical activity as part of household chores is not beneficial for work-related fatigue, whereas sport activities are (Demerouti et al. 2009). Based on these considerations, it is important that future studies measure the intensity (i.e. low, moderate, high), duration, frequency, and type (i.e. aerobic or non-aerobic, and during leisure time or part of daily life) of physical activity (e.g. Aadahl and Jørgensen 2003) and investigate which may benefit work-related fatigue most.

Third, also relating to the specific measurement of physical activity in the current study, we could not unravel whether employees’ physical activity was performed during leisure or work time. Therefore, we chose to not include employees with potentially unhealthy physical demanding work to prevent that the association between physical activity and work-related fatigue was confounded by such unhealthy physical activity. This is unfortunate, because it has been shown that employees engaging in physical demanding work can also benefit from leisure time physical activity with regard to their health (e.g. Holtermann et al. 2013). Future research could further disentangle the associations between physical activity, work-related fatigue, and task demands by explicitly examining employees with physically demanding work.

A fourth issue that needs attention is that there was relatively small across-time variation in the variables included in this study. This is reflected in the relatively high test–retest correlations over time (ranging from *r* = 0.65 to 0.69) and stable mean scores (see Table 1). This would imply that a longer time interval should be covered in order to investigate the impact of change in physical activity on work-related fatigue and task demands. Furthermore, including more time points is preferable to detect the ‘true’ time lag underlying the observed associations (Taris and Kompier 2003).

Fifth, as our sample consisted of relatively healthy workers, it may well be possible that there was a restriction of range leading to an underestimation of the true relationships found in this current study. For instance, in accordance with previous studies that demonstrated negative associations between physical activity on clinical levels of work-related fatigue (Bültmann et al. 2002; Gerber et al. 2013), the effect of physical activity on work-related fatigue found in this study might be an underestimation of the true (causal) effect. Further research is needed to investigate this.

Sixth, our selection criteria of participants resulted in a relatively small proportion of the original sample (28.8 %). Although our choice of selecting full-time (≥36 working hours) employees was based on theoretical grounds, it would be interesting to see whether the relations found in our study also exist if we would have used other working hour limits. We therefore reanalysed our data, including all employees, irrespective of their working hours. The results of these analyses (based on all employees, irrespective of their working hours) revealed a comparable pattern of results,^1^ which underlines the robustness of our findings.

Finally, we were not able to draw firm conclusions regarding causality with respect to the observed longitudinal associations, because we could not eliminate the influence of potentially relevant third variables (Taris and Kompier 2003). To get further insight into the causal associations between physical activity and work-related fatigue and task demands, further research is needed in the form of well-designed randomized controlled trials, for instance targeting physical activity levels of employees with (clinical levels of) work-related fatigue (Proper et al. 2002).

## Theoretical and practical implications

We believe our study contributes to previous research on physical activity and employee health both theoretically and practically. To our knowledge, we were among the first to longitudinally investigate ‘bi-directional’ relationships between physical activity and work-related fatigue, and physical activity and task demands. By doing so, we were able to provide a basic understanding of how physical activity is related to work-related mental health and to work characteristics. Furthermore, we tried to obtain more thorough insight into these relationships by investigating how work-related fatigue and task demands develop as a function of different (i.e. changed) meaningful physical activity patterns over time, based on employees’ compliance with the international physical activity standards (World Health Organization [WHO] 2010). Even though our core variables under study proved to be rather stable during the one-year time lag of this study, and even though physical activity could have been measured more thoroughly, we were able to demonstrate that an increase in physical activity was related to a decrease in work-related fatigue over time. This highlights the importance of physical activity for the protection of employee health and well-being. But, we also demonstrated that fatigued workers, who would benefit most from physical activity, engaged less in this type of activity. Therefore, it seems important to pay attention how to motivate fatigued employees to engage in regular physical activity. For instance, evidence shows that learning to focus on the ending of a physical activity session, instead on the often unpleasant beginning of physical activity, is a potential to increase people’s physical activity level (Ruby et al. 2011). In addition, the finding that changes in adherence to international standards of physical activity were not related to accompanying changes in work-related fatigue, indicates that relying on changes in compliance with this standard may not the most suitable way to examine changes in work-related fatigue. In this respect, it is interesting to note that the dichotomous approach of the physical activity norm is currently under discussion. Although it has been shown that the physical activity norm is certainly meaningful for (mental) health (Haskell et al. 2007), it has been argued that a slight increase in physical activity for people who are inactive could already result in health benefits (De Sauto Barreto 2015; Sparling et al. 2015). Thus, a dose–response approach (i.e. small incremental increases in daily physical activity) may be more appropriate than a ‘threshold approach’ (i.e. compliance or non-compliance) for promoting physical activity.

From a practical point of view, current findings suggest that it is valuable for employees to be physically active, in leisure time as well as during work time (Commissaris et al. 2008). Based on the demonstrated beneficial effects of physical activity, the employer can be encouraged to promote physical activity at the workplace by stimulating physically active transportation to work, designing ‘active’ workplaces or offering physical activity programs at work (Conn et al. 2009). Furthermore, employees should strive to make physical activity part of their daily routine, even when fatigued.

## Footnotes

1 Results of these analyses can be requested from the first author.
